# Phytochemical Profile and Microbiological Activity of Some Plants Belonging to the Fabaceae Family

**DOI:** 10.3390/antibiotics10060662

**Published:** 2021-06-01

**Authors:** Diana Obistioiu, Ileana Cocan, Emil Tîrziu, Viorel Herman, Monica Negrea, Alexandra Cucerzan, Alina-Georgeta Neacsu, Antoanela Lena Cozma, Ileana Nichita, Anca Hulea, Isidora Radulov, Ersilia Alexa

**Affiliations:** 1 Faculty of Veterinary Medicine, Banat University of Agricultural Sciences and Veterinary Medicine “King Michael I of Romania” Timisoara, Calea Aradului No. 119, 300645 Timisoara, Romania; dianaobistioiu@usab-tm.ro (D.O.); emiltirziu@usab-tm.ro (E.T.); viorelherman@usab-tm.ro (V.H.); alexandracucerzan@usab-tm.ro (A.C.); ileananichita@usab-tm.ro (I.N.); anca.hulea@usab-tm.ro (A.H.); 2 Faculty of Food Engineering, Banat University of Agricultural Sciences and Veterinary Medicine “King Michael I of Romania” Timisoara, Calea Aradului No. 119, 300645 Timisoara, Romania; monicanegrea@usab-tm.ro (M.N.); ersiliaalexa@usab-tm.ro (E.A.); 3 Faculty of Agriculture, Banat University of Agricultural Sciences and Veterinary Medicine “King Michael I of Romania” Timisoara, Calea Aradului No. 119, 300645 Timisoara, Romania; alinaneacsu@usab-tm.ro (A.-G.N.); cozma@usab-tm.ro (A.L.C.); isidora_radulov@usab-tm.ro (I.R.)

**Keywords:** *Melilotus officinalis*, *Coronilla varia*, *Ononis spinosa*, *Robinia pseudoacacia*, phytochemical profile, microbiological activity

## Abstract

This study aimed to investigate the chemical composition and the activity against *Staphylococcus aureus (S. aureus)* (ATCC 25923), *Streptococcus pyogenes (S. pyogenes)* (ATCC 19615), *Escherichia coli (E. coli)* (ATCC 25922), *Pseudomonas aeruginosa (P. aeruginosa)* (ATCC 27853), *Shigella flexneri (S. flexneri)* (ATCC 12022), *Salmonella typhimurium (S. typhimurium)* (ATCC 14028), *Haemophillus influenzae (H. influenza)* type B (ATCC 10211) and two fungal strains: *Candida albicans (C. albicans)* (ATCC 10231) and *Candida parapsilopsis (C. parapsilopsis)* (ATCC 22019) of the extracts obtained from *Melilotus officinalis* (MO), *Coronilla varia* (CV); *Ononis spinosa* (OS) and *Robinia pseudoacacia* (RP) (Fabaceae), and to identify the chemical compounds responsible for the antimicrobial effect against the tested strains. The extracts were obtained by conventional hydroalcoholic extraction and analyzed in terms of total polyphenols using the spectrophotometric method and by liquid chromatography (LC). The results have shown that the highest polyphenols content was recorded in the RP sample (16.21 mg gallic acid equivalent GAE/g), followed by the CV (15.06 mg GAE/g), the OS (13.17 mg GAE/g), the lowest value being recorded for the MO sample (11.94 mg GAE/g). The antimicrobial testing of plant extracts was carried out using the microdilution method. The most sensitive strains identified were: *E. coli, S. typhimurium, P. aeruginosa* and *S. pyogenes*, while protocatechuic acid, gallic acid, caffeic acid, quercetin, rutin, and kaempferol were identified as the chemical compounds responsible for the antibacterial effect. The analysis of the correlation between the chemical composition and the antimicrobial effect proved a moderate (r > 0.5) positive correlation between rosmarinic acid and *S. pyogenes* (r = 0.526), rosmarinic acid and *S. typhimurium* (r = 0.568), quercetin and *C. albicans* (r = 0.553), quercetin and *S. pyogenes* (r = 0.605). Therefore, it suggested possible antimicrobial activity generated by these chemical components. The results recommend the Fabaceae plants as promising candidates for further research to develop novel natural antimicrobial drugs.

## 1. Introduction

Despite the availability of several antibiotics and antimycotics, the treatment of patients, especially the immunocompromised ones, is still limited because of low drug potency. The emergence of resistant strains and diseases due to certain free radicals, mainly oxygen reactive species also makes treatment challenging. Together with the undesirable side effects of certain medicines, this situation is a serious medical problem, making it essential to find new sources of antibacterial and antifungal agents.

Based on the reports received through the European Antimicrobial Resistance Surveillance Network (EARS-Net), the WHO reports alarming growth rates of pathogenic microorganisms that have developed multiple resistances to common drugs [1]. Unfortunately, the pharmaceutical industry does not have the required rate of production of synthetic, allopathic drugs to cover the development of these multi-resistant organisms. This study is intended to obtain untested, natural compounds existing in the spontaneous flora, which have antimicrobial activity.

Our research investigates the chemical composition, antimicrobial, and phytochemical activity of four extracts obtained from the Fabaceae family flowers, which grow wild in Western Romania. The four selected plants were: *Melilotus officinalis* (MO) (Melilot, Sweet clover); *Coronilla varia* (CV) (Scorpion vetch); *Ononis spinosa* (OS) (Spiny restharrow), and *Robinia pseudoacacia* (RP) (Black locust).

The Fabaceae, or Leguminosae, is one of the three largest flowering plants, exceeded only by the Compositae and Orchidaceae, with an estimated ∼ 750 genera [2]. From an economic point of view, the Fabaceae family is second only to Gramineae, including many economically and medicinally important flowering plants.

The main criteria for their selection was their abundance in the wild flora in Western Romania, with all four plants being native or naturalized in our country but need further research [3]. The main activities of the four plants studied, and their references are presented in Table 1.

According to [19], *M. officinalis* exerted antimicrobial effects when containing flavonoids and various phenolic compounds, melilotin, volatile oil, mucilage, tannin, fatty acid, triterpenes, coumarin, bishydroxycoumarin, choline, and glycosides.

*Coronilla varia* (CV), or Crown vetch is a perennial invasive Fabaceae plant, an important source of phytochemicals such as polyphenols, including gallic acid and resveratrol [15].

*Robinia pseudoacacia* (RP) is one of the most well spread and naturated exotic plants [3]. The literature studies present the chemical composition of *R. pseudoacacia*, as flavonoids including robinin (kaempferol-3-*O*-ramnozilgalactozil-7-ramnozide), acacetin-7-*O*-rutoside, apigenin, diosmetin, luteolin, secundiflorol, mucronulatol, isomucronulatol, and isovestitol that are of pharmaceutical importance [20,21,22,23].

Many pathological ailments, including inflammatory diseases and infectious or microbial diseases, are caused by free radicals and their destruction [24,25]. The formation and activation of some reactive oxygen species (ROS) are some of the potentially damaging effects of oxygen. Many such species are represented by free radicals resulting from normal metabolic processes in the human body or from external sources [25].

However, the damaging effects of free radicals can be diminished by natural antioxidants, with the highest availability in plants. There being studies proving the extraordinary ability to collect radicals by various natural compounds extracted from plants.

Previous studies have focused on analyzing different plant biological activities to discover new antimicrobial agents that target free radicals. Several studies have demonstrated the antioxidant or radical properties of herbal extracts and the mechanism of action of these compounds [26,27].

It was determined that the antioxidant properties of plant extracts are attributed to their richness in isoprenoid quinones, which act as chain terminators of free radicals and as chelators ROS [25]. In addition, Gordon (1990) [28] indicated that the phenolic compounds existing in the commercial extracts act as primary antioxidants when reacting with the lipid and hydroxyl radicals to turn them into stable products. These compounds react with present metal ions, so chelates are formed; they consequently react with peroxide radicals and, in that way, stabilize these free radicals [25].

Given this, the purpose of this study was to determine which of the selected plants have antimicrobial activity and to identify the chemical components responsible for these properties.

The experimental part involved: (i) obtaining MO, CV, OS, and RP hydroalcoholic extracts; (ii) analyzing the total polyphenols content and polyphenolic profile of MO, CV, OS, and RP extracts using the LC methodology; (iii) testing the in vitro antimicrobial effect of extracts and individual polyphenols; (iv) the correlation between the analyzed parameters.

To the best of our knowledge, the local wild plant species selected for this study are being screened for the first time regarding their chemical composition and their antimicrobial effects linked to their specific composition.

## 2. Results

### 2.1. Chemical Composition of Extracts

**Figure 1:**
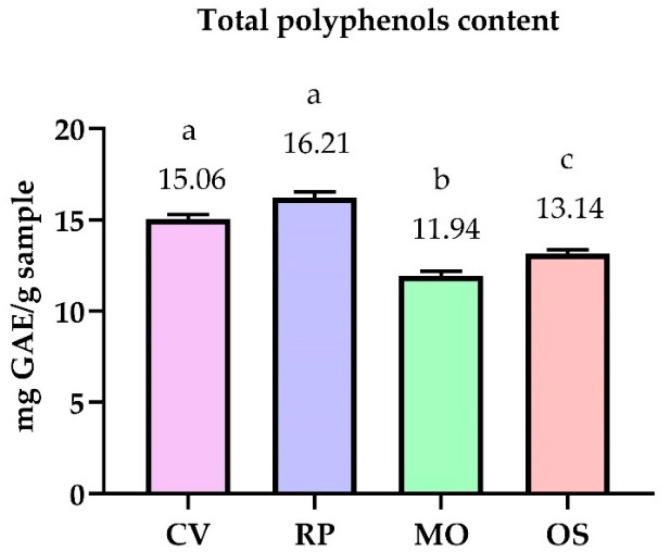
Content of total polyphenols in the studied samples. Mean values are expressed as mg gallic acid equivalent GAE/g sample. The error bars indicate standard deviation. Different letters among samples indicate significant differences (*p* < 0.05) among values according to the *t*-test.

Figure 1 presents the total polyphenols content (TPC) expressed as mg gallic acid equivalent GAE/g sample of the analyzed hydroalcoholic extracts detected using the UV spectrophotometric method. In contrast, Table 2 presents the individual profile of polyphenols identified and quantified using high-performance liquid chromatography (HPLC).

The values for total polyphenols vary within minimal limits. From the data presented, it can be seen that the highest value was recorded in the RP alcoholic extract (16.21 mg GAE/g), followed by CV (15.06 mg GAE/g), OS (13.17 mg GAE/g), and the lowest value was recorded in the MO (11.94 mg GAE/g).

The four investigated Fabaceae species revealed distinct chemical patterns (Table 2). In the MO alcoholic extract, the main components were: epicatechin (65.879 mg·g^−1^), kaempferol (1.114 mg·g^−1^), rutin (7.865 mg·g^−1^), and caffeic acid (2.441 mg·g^−1^). Smaller quantities of protocatechuic acid (0.696 mg·g^−1^), coumaric acid (0.999 mg·g^−1^), resveratrol (1.518 mg·g^−1^), and rosmarinic acid (0.64 mg·g^−1^) were also detected.

Our data showed that in case of the RP alcoholic extract, the main polyphenolic components were rutin (35.257 mg·g^−1^), epicatechin (17 mg·g^−1^), rosmarinic acid (4.439 mg·g^−1^), and resveratrol (2.175 mg·g^−1^). Other compounds found in minority were gallic acid (0.693 mg·g^−1^), quercetin (1.786 mg·g^−1^), protocatechuic acid (0.701 mg·g^−1^), caffeic acid (0.567 mg·g^−1^), and kaempferol (0.669 mg·g^−1^).

The CV extract presented as primary polyphenolic components the following: rutin (2.779 mg·g^−1^), epicatechin (2.219 mg·g^−1^), rosmarinic acid (2.051 mg·g^−1^), kaempferol (1.878 mg·g^−1^), gallic acid (0.249 mg·g^−1^), and resveratrol (1.256 mg·g^−1^); in the OS extract: kaempferol (4.861 mg·g^−1^), rosmarinic acid (0.043 mg·g^−1^), quercetin (2.838 mg·g^−1^), and rutin (2.156 mg·g^−1^).

### 2.2. Antimicrobial Activity

**Figure 2:**
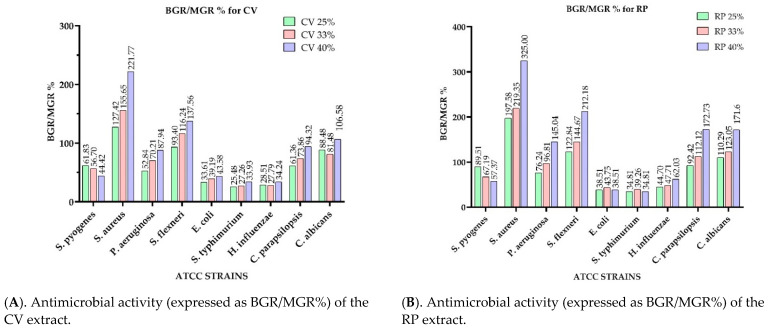

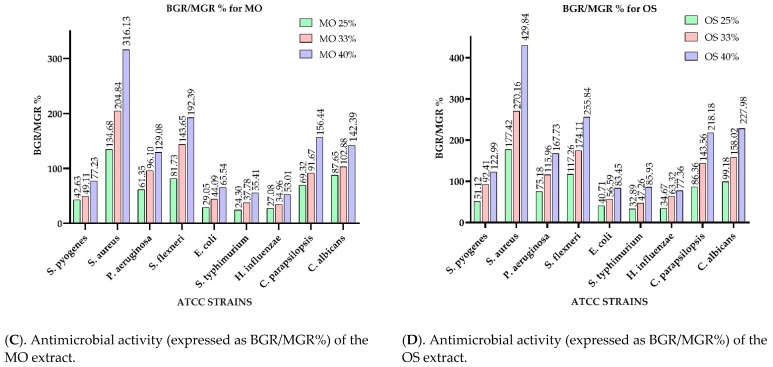
Antimicrobial activity of analyzed extracts expressed as BGR/MGR (%). (**A**). CV extract; (**B**). RP extract; (**C**). MO extract; (**D**). OS extract.

**Figure 3:**
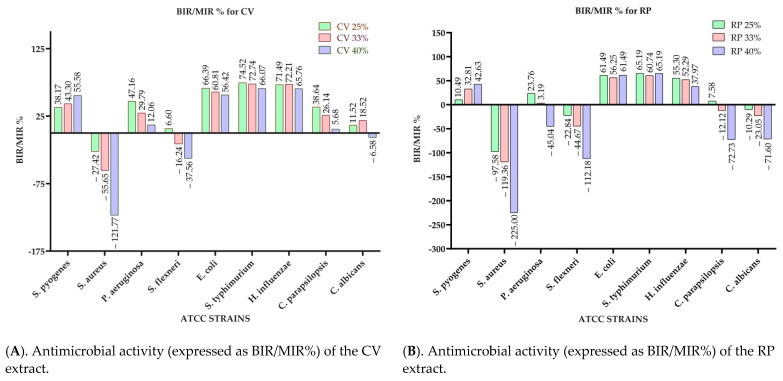

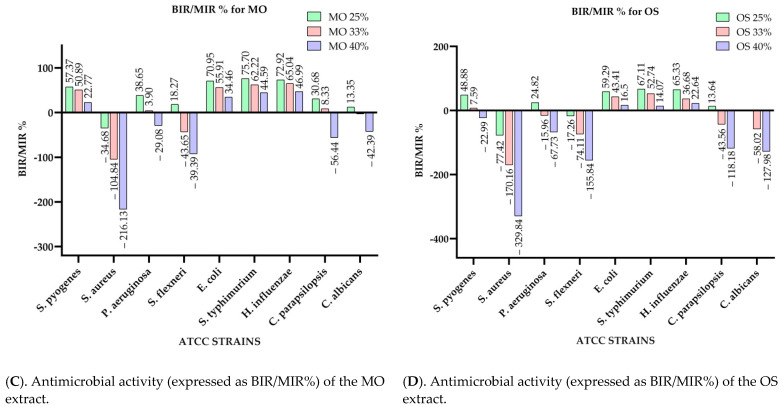
Antimicrobial activity of extracts expressed as BIR/MIR (%) calculated according to Formula (1). (**A**). CV extract; (**B**). RP extract; (**C**). MO extract; (**D**). OS extract.

Figure 2 presents the microdilution method results of the analyzed extracts, expressed as bacterial (for bacteria-BGR%) or mycelial growth rate (in case of fungi-MGR%), calculated according to Formula (1). Meanwhile, Figure 3 shows the bacterial inhibition rate (BIR %)/mycelial inhibition rate (MIR %), calculated according to Formula (2).

Two indicators were calculated, BGR for MGR and BIR/MIR, to interpret the results, using the following formulas:

(1) $$\mathrm{BGR}/\mathrm{MGR}=\frac{OD_{sample}}{OD_{negativecontrol}}\times 100\left(\%\right)$$

BIR/MIR = 100 − BGR/MGR (%)(2)

where OD sample—is the optical density at 540 nm as a mean value of triplicate readings for extracts and standards in the presence of selected strains; OD negative control—the optical density at 540 nm as a mean value of triplicate readings for the selected strains in BHI.

A table containing the OD (optical density) values when different concentrations of extracts were applied on the screened strains is presented in the Supplementary Table S1.

Comparing the BGR percentages, the most sensitive ATCC strains, in the case of the CV extract, were: *H. influenzae, S. typhimurium, E. coli, and S. pyogenes* (Figure 2A). For *H. influenzae,* the bacterial growth rate (BGR%), depending on the concentration of the extract tested (CV 25%; CV 33% and CV 40%), varied from 28.51–34.24% (Figure 2A), with a bacterial inhibition rate (BIR%) ranging from 65.76–71.49% compared to the negative control (Figure 3A), while the BIR against *S. typhimurium* was 66.07–74.52%.

The concentration influence trend was similar for the other sensitive strains. In CV, BIR ranged between 38.17%–55.58% against *S. pyogenes,* and between 56.42% and 66.39% against *E. coli*. Regarding the antifungal activity, the fungal inhibition rate showed a higher effect on *C. parapsilopsis* than on *C. albicans*, values being different by 20%.

*S. aureus* and *S. flexneri* were the only two ATCC strains on which the inhibitory effect of CV was not observed. The evolution of BIR% in their case was negative, proving a bacterial boosting effect. The growth rate for *S. aureus* was concentration-dependent, ranging from 127.41% to 221.77%. The bacterial growth effect on *S. flexneri* was demonstrated in the case of CV by BGR% values ranging from 93.4% to 137.56% (Figure 2A).

To summarize the data regarding the RP antimicrobial activity presented in Figure 2B and Figure 3B, RP was the most effective against *S. pyogenes, E. coli, S. typhimurium,* and *H. influenzae* with BIR values between 7.58% and 65.19% (Figure 3B). Regarding the other tested strains *(S. aureus, P. aeruginosa, S. flexneri*, and both *Candida* strains), RP had a proven growth-boosting effect. It had a BGR compared to control (%) that varied from 197.58%–325% for *S. aureus*, 76.24%–145.04% for *P. aeruginosa*, 122.84%–212.18% for *S. flexneri*, and 92.42%–172.73% MGR for *C. parapsilopsis,* and 110.29%–171.60% for *C. albicans*. It was noted that the boosting effect was influenced by the quantity of the extract tested, MGR growing together with the concentration (Figure 2B).

Concerning the three concentrations of the MO alcoholic extracts (MO 25%, MO 33%, and MO 40%), the results presented in Figure 2C and Figure 3C show the best antimicrobial effect recorded against *S. pyogenes* (with BGR ranging from 42.63%–77.23%, and BIR 22.77%–57.37%), *E. coli* (with BGR values of 29.05%–65.54%, and BIR of 34.46%–70.95%) and *S. typhimurium* (BGR of 24.30%–55.41%, BIR of 46.99%–72.52%), depending on the concentration.

The boosting effect was linked to the concentration of the extracts. BGR% ranging from 134.68% for MO 25% up to 316.13% for MO 40% for *S. aureus*, 61.35% for MO 25% up to 129.08% for MO 40% on *P. aeruginosa,* with values ranging from 81.73% for MO 25% and 192.39% for MO 40% for *S. flexneri.* Meanwhile, for both fungal strains, the percentages ranged from 69.32% in MO 25% up to 156.44% for MO 40% (Figure 2C).

Regarding the effect of all three OS extracts tested (OS 25%, OS 33% and OS 40%), the data obtained revealed a boosting effect, proved by the negative values of BIR on *S. pyogenes*, with BIR% values ranging from −22.99% when OS 40% was applied, −77.42% to −329.84% on *S. aureus*, −15.96% to −67.73% on *P. aeruginosa*, *S. flexneri* −17.26% to −155.84%, and both fungal strains with MIR% ranging from −13% down to −127%. The inhibitory effect of the OS extract was proven against *E. coli* (MIR ranging from 16.25%–59.29%*, S. typhimurium* with BIR 67.11%–14.07%, and *H. influenzae* with 65.33%–13.64%. All the results for BGR/MGR% and BIR/MIR% values when the OS extract was used are presented in Figure 2D and Figure 3D. The statistical analysis performed in Table 3 highlighted significant differences between the antimicrobial activity against the tested strains depending on extract matrices and concentration.

After the antimicrobial assays of extracts, the chemical compounds responsible for the antimicrobial effect were tested individually. In this regard, standard chemical substances identified by LC as the main polyphenol compounds in the extracts were tested on the selected four sensitive strains, eliminating the bacteria and fungi that proved to be unaffected by the tested extracts.

Table 3 presents the bacterial growth rate (BGR%) and the bacterial inhibition rate (BIR%) of individual polyphenol standards at 50 and 500 mg·g^−1^.

The MICs (µL/100 mL) values, defined as the lowest concentration of the compounds to inhibit the growth of microorganisms, CV, RP, MO, and OS extracts and chemical compounds, are presented in Table 4 and Table 5.

According to the data presented in Table 4, no inhibition effect, causing a mass growth (marked in dark grey color) regardless of the concentration tested, was observed against S. *aureus.* Regarding the most extracts and concentrations tested, the MIC value varied between 25–40 µL/100 mL, but subsequent concentrations showed a potentiating effect. Therefore, the effect decreased together with the concentration. The MIC of the CV and RP extract against *S. pyogenes*, respectively, and the MO extracts against *P. aeruginosa* was 25 µL/100 mL. The effect was maintained with an increase in concentration (white color).

Regarding the polyphenols standards, the MIC of gallic acid, ferulic acid, and resveratrol was 50 mg·g^−1^ against *E. coli*. However, the effect decreased together with the concentration. The values tested were not the correct quantity to define MIC in the case of the coumaric acid. For the rest of the standards, namely protocatechuic acid, caffeic acid, epicatechin, rutin, rosmarinic acid, quercetin, and kaempferol, 50 mg·g^−1^ proved to be the MIC. The effect was maintained together with an increase in concentration (Table 5).

For *S. typhimurium,* the results showed no effect for coumaric and ferulic acids, potentiating for gallic and caffeic acids, and MIC at 50 mg·g^−1^ when protocatechuic acid, epicatechin, rutin, rosmarinc acid, resveratrol, quercetin, and kaempferol were used.

The results on *P. aeruginosa* showed that only ferulic and rosmarinic acids did not affect at 50 and 500 mg·g^−1^. Other polyphenols presented an MIC at 50 mg·g^−1^.

Regarding the effect of the standards against *S. pyogenes*, gallic acid and resveratrol had a strain-boosting effect, while coumaric, ferulic, and rosmarinic acids had no effect. Epicatechin, rutin, quercetin, kaempferol, and protocatechuic acid proved an inhibitory effect on all tested strains, and the MIC was 50 mg·g^−1^.

The analysis of correlation (Table 6) between the chemical composition and the antimicrobial effect of the plant extracts against the analyzed strains proved a moderate (r > 0.5) positive correlation between the pairs. Rosmarinic acid and *S. pyogenes* (r = 0.526)*,* rosmarinic acid and *S. typhimurium* (r = 0.568), quercetin and *C. albicans* (r = 0.553), quercetin and *S. pyogenes* (r = 0.605), suggesting possible antimicrobial activity generated by these chemical components.

Other strong (r > 0.7) positive correlations were recorded between the following pairs:

- S. pyogenes and S. typhimurium (r = 0.914), H. influenzae (r = 0.817), C. parapsilopsis (r = 0.715), C. albicans (r = 0.795).
- S. aureus and P. aeruginosa (r = 0.984).
- S. flexneri and S. typhimurium (r = 0.720), H. influenzae (r = 0.936), C. parapsilopsis (r = 0.988), C. albicans (r = 0.920).
- S. typhimurium and H. influenzae (r = 0.777), C. parapsilopsis (r = 0.728), C. albicans (r = 0.737).
- H. influenzae and C. parapsilopsis (r = 0.958), C. albicans (r = 0.946).
- C. parapsilopsis and C. albicans (r = 0.941).
- Gallic acid and rutin (r = 0.896), resveratrol (r = 0.848).
- Protocatechuic acid and epicatechin (r = 0.750), resveratrol (r = 0.826).
- Caffeic acid and epicatechin (r = 0.959), coumaric acid (r = 0.986), kaempferol (r = 0.943).
- Epicatechin and coumaric acid (r = 0.990), kaempferol (r = 0.824).
- Coumaric acid and kaempferol (r = 0.874).
- Ferulic acid and quercetin (r = 0.810); rutin and resveratrol (r = 0.977).
- Rosmarinic acid and quercetin (r = 0.930).
- A strong negative correlation was highlighted between the following pairs:
- Gallic acid and kaempferol (r = −0.753); protocatechuic acid and ferulic acid (r = −0.703); caffeic acid and rosmarinic acid (r = −0.794; epicatechin and rosmarinic acid (r = −0.717); coumaric acid and rosmarinic acid (r = −0.790); coumaric acid and quercetin (r = −0.706).

## 3. Discussion

### 3.1. Chemical Composition of Extracts

Other authors previously studied the polyphenolic content of the RP and MA samples, and the results were falling within the range of values obtained in this study. Thus, in the case of RP, Marinas et al. (2014) [29] found a content of 26.67 mg GAE/g, and for MA, the level was reported at 14.8 mg GAE/g [30]. Higher values of phenolic compounds in the flowers (0.77 mg GAE/mL) were observed compared to syrup 60° Brix (0.06 mg GAE/mL) and *R. pseudoacacia* syrup 70° Brix (0.14 mg GAE/mL) [31]. Serbian RP showed values of 74.28 ± 1.73 for TPC (mg GAE/g) [29], while Arnold et al. (2015) [32] reported a phenolic content of 20.55 ± 2.56 mg GAE/g in the OS extract.

Previous studies showed that *M. officinalis* L., contained coumarins, flavonoids, steroids and saponins, phenolic acids, volatile components, fats, alcohols, uric acid, and other chemical compounds [33,34,35]. These compounds have antiinflammatory, swelling, and antitumour properties, as well as therapeutic effects against hemorrhoids, thrombophlebitis, and varicose veins [33,36,37,38].

Another study in Poland reported the following key compounds in the MO extract: coumaric acid (0.443 mg·g^−1^), protocatechuic acid (0.149 mg·g^−1^), ferulic acid (0.444 mg·g^−1^), and caffeic acid (0.849 mg·g^−1^), while the gallic acid was found in a concentration of 0.119 mg·g^−1^ [39]. In an ethanol 96% extract, Molnar et al. (2017) [40] found the coumarin concentration in whole *M. officinalis* to be 3.163 mg·g^−1^. In an ethanol 50% extract, the concentration was 1.464 mg·g^−1^. In a review, Al-Snafi et al. (2020) [41] cited Safapour et al. (2015) [8] concerning HPLC analysis that revealed the flower powder of MO contained 9.7 mg·g^−1^ gallic acid, 99 mg·g^−1^ catechin, 21.9 mg·g^−1^ caffeic acid, 0.86 mg·g^−1^ chlorogenic acid, 1.13 mg·g^−1^ quercetin, 548.9 mg·g^−1^ cinnamic acid, 289 mg·g^−1^ coumarin, and 126 mg·g^−1^ *p*-coumaric acid. This research was also confirmed by Jasicka-Misiak et al. (2017) [42].

Our findings are in accord with Călina et al. (2013) [43], who proved that flavonoids, such as rutin (ruthoside) and hyperoside were found in methanolic extracts from flowers, leaves, bark, and seeds of RP from western Romania. Their findings showed that the flower extract contained more hyperoside (0.9 mg·g^−1^) than the leaf extract (0.17 mg·g^−1^). On the other hand, in rutin, the leaves contained almost six times more rutin than the flowers (0.98 mg·g^−1^ vs. 0.17 mg·g^−1^). The literature also cites the chemical content of RP wood in gallic acid with values ranging from 27–296 mg·g^−1^ [43]. The same two primary components were found in the CV extract, the difference being the smaller concentration. According to the literature, the main bioactive components found in the RP flowers include flavonoids, phenolics, ascorbic acid, polysaccharide, and some microelements [44,45,46].

Another study performed in Serbia [47] showed gallic acid in RP (58.74 mg·g^−1^).

Ferrante et al. (2020) [15] studied the OS extracts from a chemical point of view and found that the main polyphenols identified were gallic acid (1.17 mg·g^−1^), catechin (13.06 mg·g^−1^), epicatechin (1.4 mg·g^−1^), and rutin (2.92 mg·g^−1)^. They appeared in almost similar ranges to our findings.

According to the literature, the main bioactive components found in Acacia flowers include flavonoids, phenols, ascorbic acid, polysaccharides, and some trace elements. Ferrante et al. (2020) [48] made a study on two types of OS extracts and discovered that in case of the hydroalcoholic extract, the gallic content was 227.25 ± 9.11 mg·g^−1^. Meanwhile, the resveratrol content was 61.82 ± 6.99 mg·g^−1^. The data presented in their study support our findings.

### 3.2. Antimicrobial Activity

Regarding the antimicrobial effect on the ATCC 19615 strain of *S. pyogenes*, the OS extract was the most effective while the CV was the least effective. The OS and RP demonstrated an inhibitory effect on the growth of the *S. pyogenes* mass, while MO and CV proved a strain-boosting effect. Rosu et al. (2012) [49] showed the antimicrobial effect of the RP extracts obtained from different plant parts against *P. aeruginosa*. Ferrante et al. (2020) [15] demonstrated the lack of antibacterial effect of the OS extract on *S. pyogenes*.

Our results on the ATCC strain of *S. aureus* showed the following classification: MO > RP > CV > OS. MO and RP have demonstrated an inhibitory effect, and CV proved a strain-boosting effect with a mycelial growth rate, BGR (%), up to 223.96%. The same effect was present in the case of OS, with a mycelial growth rate BGR (%) up to and 442.97%, values presented in Figure 2. These findings aligned with research by Marinas et al. (2014) [29], which proved that RP had an inhibitory effect on *S. aureus* ATCC and MRSA strains, while Sisay et al. (2019) [50] showed the effect of *M. elegans* against *S. aureus*. Ferrente et al. (2020) [15] demonstrated a medium effect on *S. aureus*, with a MIC of 99.21 µg/mL in the case of the OS extract.

*P. aeruginosa* was inhibited by MO and RP. At the same time, CV and OS stimulated its growth (MO ↓ > RP ↓ > CV ↑ > OS ↑), with values relative to the negative control. Other studies reported the same findings on RP [48,50] and OS [51] against *P. aeruginosa.* Karakas et al. (2012) [52] proved an inhibitory effect of the MO extract, which would justify the use of MO in folk medicine as part of inflammation-related therapy (caused by *P. aeruginosa*).

The development of *E. coli* colonies was inhibited by RP and OS and was stimulated by CV and MO (RP ↓ > OS ↓ > CV ↑ > MO ↑). The good antibacterial effect against *E. coli* was also demonstrated [30,32] for RP and MO [50]. In the case of MO, a medium effect on *E. coli* was observed [51], while our studies are supported by Ferrente et al. (2020) [15], who proved a promising effect of OS on three *E. coli* strains, with concentrations ranging from 31–250 µg/mL.

The analyzed extracts had no inhibitory effect on *S. flexneri* (MO ↑ > CV ↑ > RP ↑ > OS ↑). This finding is supported by Sisay et al. (2019) [50].

*S. aureus* was not inhibited by the studied extracts. All extracts had a colony-stimulating action (RP ↑ > CV ↑ > MO ↑ > OS ↑), being one of the few microorganisms more difficult to control [52]. In the cited literature [53], MO proved a weak effect on *S. aureus*, RP showed no effect on *S. cholerae* in any of the four types of extracts tested, and OS proved no effect against *S. aureus* (PeruMycA 7) in concentrations higher than 250 µg/mL [1,29,31].

All the extracts had the effect of stimulating the growth of *H. influenzae* and *C. parapsilopsis,* and therefore, we conclude that the effect was a boosting one (*H. influenzae*: CV ↑ > MO ↑ > RP ↑ > OS ↑; *C. parapsilopsis*: CV ↑ > MO ↑ > RP ↑ > OS ↑).

On *C. albicans* colonies, only the CV extract had an inhibitory effect, with all the other extracts stimulating the fungal mass development (*C. albicans*: CV ↓ > MO ↑ > RP ↑ > OS ↑). The same effect was noted, according to Ferrente et al. (2020) [48], on *C. albicans* (YEPGA 6183), with values ranging from 7–11 µg/mL. In other studies, RP proved no effect either [49].

Our findings showed that the efficacy of the extracts and standards tested, expressed as strain mass loss or mass growth, was closely correlated with the concentration.

In terms of efficacy on *S. pyogenes*, regarding the standard polyphenols (Table 3), the BGR% values classification was caffeic acid 49% (present in MO, OS) > kaempferol 52% (present in MO, OS) > protocatechuic acid 53% (present in RP, MO) > epicatechin 61% (present in MO, RP) > quercetin 63% (present in OS, RP) > rutin 66% (present in RP).

According to this phase of our research, we may assume that the effectiveness of the OS extract is given by the amount of caffeic acid, kaempferol, quercetin.

Our results on the ATCC *S. typhimurium* proved, from the point of view of the antimicrobial efficacy of the extracts, the following classification: CV > MO > RP > OS.

According to the data presented in Table 3, regarding the efficacy of the extracts on *S. typhimurium*, the most significant effect was shown by protocatechuic acid (MO) > gallic acid (RP, CV)> caffeic acid (MO) > quercetin (OS) > rutin (MO, RP, CV) > kaempferol (OS, MO, CV). These results correlated with the results obtained in the first part of our study, which emphasized the antibacterial effect of the extracts against *S. typhimurium*, with the data presented in Table 3.

Regarding the efficacy of the selected polyphenols on *P. aeruginosa*, we observed that the greatest inhibition was proved by kaempferol (MO, OS). It was followed by protocatechuic acid (MO, RP), caffeic acid (MO, OS), epicatechin (MO, RP), gallic acid (RP ↑ with concentration), rutin (RP), and quercetin (OS).

MO had in its composition the following polyphenols with antimicrobial activity on *P. aeruginosa*: kaempferol, protocatechuic acid, caffeic acid, and epicatechins.

The development of *E. coli* colonies was inhibited by RP and OS and was stimulated by CV and MO (RP ↓ > OS ↓ > CV ↑ > MO ↑).

On the development of *E. coli,* the most effective inhibitor was protocatechuic acid 49% BGR (MO, RP). This was followed by kaempferol 51% BGR (MO, OS), caffeic acid 59% BGR (MO, OS), epicatechins 58% BGR (RP, MO), gallic acid 63% BGR (RP) (↑ with concentration), and rutin 77% BGR (RP), as presented in Table 3.

According to Table 2, RP had the following polyphenols with antimicrobial activity on *E. coli*: protocatechuic acid, epicatechins, quercetin, and rutin. OS contained caffeic acid, kaempferol, and quercetin with an inhibitory effect on *E. coli*.

Macé et al. (2017) [53] used the microdilution method to test the effectiveness of several polyphenolic compounds at a concentration of 100 µg mL^−1^ on *S. pyogenes*. They found that resveratrol was the most active from the list of standards that we tested. Meanwhile, epicatechin, quercetin, gallic acid, and ferulic acid were used only in the preliminary tests, and they proved no interfering effect on bacterial growth. Abachi et al. (2015) [54] also proved the inhibitory effect of epicatechin on *S. pyogenes*.

Bouarab-Chibane et al. (2019) [55] proved the antimicrobial effect of caffeic acid, rutin, epicatechin, and resveratrol against the tested *P. aeruginosa*, while, in their study, quercetin showed no effect. The researchers explained the mechanisms of antibacterial action of phenolic compounds at the cellular level, involving the modification in permeability of the cell membranes, the changes in various intracellular functions induced by hydrogen bonding of the phenolic compounds to enzymes, or by the modification of the cell wall rigidity with integrity losses due to different interactions with the cell membrane [55,56,57,58]. These processes induce irreversible damage of the cytoplasmic membrane, phenolic acids responsible for the disruption of the membrane integrity, causing leakage of the essential intracellular constituents. In the case of Gram-positive bacteria, intracellular pH modification and interference with the energy (ATP) were reported [54,59,60,61].

Marinas et al. (2014) [29], in their experiments, showed that alcoholic acacia extracts have antimicrobial activity against both *Candida*, Gram-positive bacteria (*S. aureus, Bacillus subtilis,* and *Enterococcus faecalis*) and Gram-negative (*P. aeruginosa, E. coli, Klebsiella pneumoniae,* and *Acinetobacter baumannii*).

Talas Ogras et al. (2005) [62] studied the in vitro antibacterial activity of the extract from *R. pseudoacacia* seeds, noting that *S. aureus* showed the highest sensitivity to the action of the extract.

According to the literature, it has been observed that several researchers have confirmed the antimicrobial activity of *M. officinalis*. Aćamović-Đoković et al. (2002) [63] compared the activity of *M. albus* (white sulphine), *M. melissophyllum,* and *M. officinalis* (yellow sulphine). Their study tested the antibacterial activity of petrol ether and ethyl acetate extract using the disk diffusion method in relation to *E. coli*, *P. mirabilis*, *Salmonella enteritidis*, *P. aeruginosa*, *Streptococcus-haemoliticus* type A, *S. aureus*, and *C. albicans*. The MO was more efficient than *M. albus* extracts.

Karakas et al. (2012) [52] carried out comparative research on biological activities and examined 16 plants from Turkey, including *M. officinalis*, using the diffusimetric method. The antibacterial activity of the aqueous extract of *M. officinalis* showed an area of growth inhibition on the strain tested on *P. aeruginosa* of 22.5 mm. Moreover, methanol and ethyl acetate extracts from *M. officinalis* proved an inhibitory effect on fungi.

The antifungal activity of various *M. officinalis* extracts was tested on 12 pathogenic plant fungi in vitro and in vivo. The results showed that the antifungal activity of the ethyl acetate extract from *M. officinalis* was higher than the activity of the ethyl acetate extracts from other plants [64].

Studies on the effect of the ferulic and gallic acids on the mass growth of *E. coli, P. aeruginosa, S. typhimurium,* and *L. monocytogenes* were conducted by Borges et al. (2013) [60]. Protocatechuic acid showed an inhibitory effect on methicillin-resistant colonies of *S. aureus*, *Klebsiella pneumonia, P. aeruginosa,* and *A. baumannii* [65].

Resveratrol had an antibacterial effect against pathogenic bacteria in food: *S. aureus, E. coli* O157: H7, *S. typhimurium,* the action being more intense on Gram-positive than Gram-negative strains [66,67].

The literature structured on this topic proves the benefit of using natural products as microorganisms cannot acquire resistance to all biological compounds [68,69,70,71,72]. This becomes a new, viable option to fighting new and increasing strain resistance. Moreover, a great benefit of using natural products is the possible synergism or antagonism obtained due to the great diversity concerning the extract/oil content.

## 4. Materials and Methods

### 4.1. Plant Material

The aerial parts of the investigated medicinal plant species (MO, CV, OS, and RP) were collected during the flowering period from the wild flora located on the outskirts of Timisoara, Romania (45°47′00.1″ N, 21°12′37.2″ E) in 2018. From each species, ~500 g of fresh material was used. Additionally, voucher specimens were botanically identified and deposited in a temperature-controlled herbarium (22–25 °C and 30–40% relative humidity) in the Botany Department, at the Banat University of Agricultural Sciences and Veterinary Medicine ‘King Michael I of Romania’ in Timişoara (Vouchers Specimen Number Herbarium-Botany Department, M.O-VSNH.BUASTM-BD56, C.V-VSNH.BUASTM-BD57, 0.S-VSNH.BUASTM-BD58, R.P-VSNH.BUASTM-BD59).

### 4.2. Preparation of Extracts

The plant material was air-dried at 25 °C and ground to a fine powder using a grinder (GM 2000; Grindomix; Retsch Technology GMbH, Haan, Germany). The powdered material (2 g) was extracted with 20 mL 60% ethanol (Sigma-Aldrich; Merck KGaA, Darmstadt, Germany) for 30 min at room temperature using an ultrasonic water bath (FALC Instruments, Treviglio, Italy) [73]. Extracts were then filtered using Whatman membrane filters nylon 0.45 µm with 30 mm diameter (Sigma-Aldrich; Merck KGaA, Darmstadt, Germany) and stored at 2–4 °C for subsequent chemical and antimicrobial analyses [73].

### 4.3. Determination of Total Polyphenols Content by Folin-Ciocalteu Assay

The total phenolics content was determined according to Folin–Ciocalteu modified method [73]. An amount of 0.5 mL extract was treated with 1.25 mL Folin-Ciocalteu reagent (Sigma-Aldrich Chemie GmbH, München, Germany) diluted 1:10 with distilled water. The sample was incubated for 5 min at room temperature, then 1 mL Na_2_CO_3_ (Geyer GmbH, Renningen, Germany) (60 g/L aqueous salts) was added. The samples were incubated for 30 min at 50 °C in an INB500 thermostat, Memmert GmbH, Schwabach, Germany) after reading absorbance at 750 nm using a UV-VIS spectrophotometer (Specord 205; Analytik Jena AG, Jena, Germany). As a reference, ethanol (Sigma-Aldrich; Merck KGaA, Darmstadt, Germany) was used. The calibration curve was obtained using gallic acid (concentration range: 2.5–250 μg/mL). The results were expressed in mg GAE per g of dry matter (d.m.). All determinations were performed in triplicate.

### 4.4. Determination of Individual Polyphenols by LC Analysis

LC analysis was performed using a Shimadzu chromatograph (Shimadzu 2010 EV, Kyoto, Japan) equipped with an SPD-10A UV detector, EC 150/2 NUCLEODUR C18 Gravity SB 150 × 2 mm × 5 μm column (Macherey-Nagel GmbH & Co. KG, Dueren, Germany). The chromatographic system comprised an LC unit with a UV-VIS spectrophotometer detector (SPD-10A), a degasser, an autosampler, and solvent delivery pumps (LC-10AD). Chromatographic conditions were as follows. Mobile phases A: water acidified with formic acid (Merck KGaA, Darmstadt, Germany) at pH-3, and B: acetonitrile (Merck KGaA, Darmstadt, Germany) acidified with formic acid at pH-3. The gradient program was 0.01−20 min 5% B, 20.01−50 min 5−40% B, 5−55 min 40−95% B, 55−60 min 95% B. Solvent flow rate of 0.2 mL/min, temperature 200 °C. The monitoring wavelength was 280 nm and 320 nm. The calibration curves were performed in the range of 20−50 μg/mL. The calibration curves were produced in the range of 1–10 μg/mL. The results were expressed in mg·g^−1^ d.m. The experiments were performed in triplicate. All standards were prepared in methanol (Merck KGaA, Darmstadt, Germany) and all reagents and solvents used were analytical grade chemicals. Standards were purchased from Sigma-Aldrich, Merck KGaA, Darmstadt, Germany.

### 4.5. Antimicrobial Activity

The extracts were tested against *S. aureus* (ATCC 25923), *S. pyogenes* (ATCC 19615), *E. coli* (ATCC 25922), *P. aeruginosa* (ATCC 27853), *S. flexneri* (ATCC 12022), *S. typhimurium* (ATCC 14028), *H. influenzae* type B (ATCC 10211), *C. albicans* (ATCC 10231), and *C. parapsilopsis* (ATCC 22019).

The microorganisms used in this study were obtained from the culture collection of the Laboratory of Microbiology in the Interdisciplinary Research Platform within Banat’s “King Michael I of Romania” the University of Agricultural Science and Veterinary Medicine Timisoara. In our laboratory, the ATCC strains were maintained at −50 °C.

McFarland standards were used to approximate the concentration of cells in a suspension visually. The McFarland scale is CFU/mL specific concentrations and is designed to be used to estimate bacterial concentrations [74].

#### 4.5.1. Bacterial Culture

A 10^−3^ dilution of the fresh culture was used to perform the assay, an inoculum equivalent to a 0.5 McFarland standard. The ATCC microorganisms were revived by overnight growth in Brain Heart Infusion (BHI) broth (Oxoid, CM1135), at 37 °C, and subsequently, passed on BHI Agar (Oxoid, CM1136), for 24 h at 37 °C. The cultures were then diluted at an optical density (OD) of 0.5 McFarland standard (1.5 × 10^8^ UFC × mL) using BHI broth. The suspensions were tested using a 96 microdilution well plate. Using a Calibra digital 852 multichannel pipette, 100 μL of microbial suspension was placed in each well. The extracts were used directly, placing either 25, 50, or 100 μL in each well. The plates were covered and left 24 h at 37 °C. After 24 h, the OD was measured at 540 nm using an ELISA reader (BIORAD PR 1100, Hercules, CA, USA). Triplicate tests were performed for all samples. A mixture of 60% alcohol (according to the procedure used in the preparation of extracts) was tested for an inhibitory effect. The results proved no effect on the tested strains; therefore, the results were not presented here. The suspensions of strain and BHI were used as a negative control.

The MIC is defined as the lowest compound concentration that yields no visible microorganism growth. The method of MIC determination based on the microbial mass loss by measurement of OD by spectrophotometry according to ISO 20776-1:2019 was described in our previous research [73]. To interpret the results, two indicators were calculated, BGR and BIR, using the following formulas:

(3) $$\mathrm{BGR}=\frac{OD_{sample}}{OD_{negativecontrol}}\times 100\left(\%\right)$$

BIR = 100 − BGR (%)(4)

where: OD sample—optical density at 540 nm as a mean value of triplicate readings for extracts and standards in the presence of the selected bacteria; OD negative control—optical density at 540 nm as a mean value of triplicate readings for the selected bacteria in BHI.

Concerning the standards tested, the method used was identical to the one used for the extract analysis. The quantities tested were calculated as the minimum and maximum amount of standard contained in the extracts within the same amount of extract tested, being 50 and 500 mg·g^−1^.

#### 4.5.2. Fungi Culture

A 10^−2^ dilution of the fresh culture was used to perform the assay, an inoculum equivalent to a 0.5 McFarland standard. The ATCC microorganisms were revived by overnight growth in brain heart infusion (BHI) broth (Oxoid, CM1135), at 37 °C, and subsequently, passed on BHI Agar (Oxoid, CM1136), for 48 h at 37 °C. The cultures were then diluted at an OD of 0.5 McFarland standard using BHI broth. The suspensions were tested using a 96 microdilution well plate by placing 100 μL of microbial suspension in each well. The extracts were used directly, placing 25, 50, or 100 μL in each well. The plates were covered and left for 48 h at 37 °C. After 48 h, the OD was measured at 540 nm. Triplicate tests were performed for all samples.

To interpret the results, two indicators have been calculated, MGR and MIR, using the following formulas:

(5) $$\mathrm{MGR}=\frac{OD_{sample}}{OD_{negativecontrol}}\times 100\left(\%\right)$$

MIR = 100 − MGR (%)(6)

where: OD sample—optical density at 540 nm as a mean value of triplicate readings for extracts and standards in the presence of the selected fungi; OD negative control—optical density at 540 nm as a mean value of triplicate readings for the selected fungi in BHI.

### 4.6. Statistical Analysis

All determinations were made in triplicate, and the results were reported as mean values ± standard deviation (SD).

For total and individual polyphenols content, all replicates’ mean values and standard deviations were calculated using GraphPad Prism (v.5.0 software, Manufacture, San Diego, CA, USA). The differences between means were analyzed with a one-way ANOVA, followed by a multiple comparison analysis using the *t*-test (two-sample assuming equal variances). The differences were considered significant when *p*-values < 0.05. Correlations between variables were performed using Microsoft Excel 2010.

## 5. Conclusions

The study conducted on the antimicrobial potential of plant extracts belonging to the Fabaceae family pointed out that they can be considered promising antimicrobial agents. They open a new pathway for further research to find new complementary antibiotics against Gram-positive and/or negative bacteria and antifungal agents. The most sensitive tested plant extracts proved to be CV and RP against *S. pyogenes*, respectively, and MO against *P. aeruginosa*. In terms of chemical compounds responsible for the antimicrobial effect, kaempferol, quercetin, epicatechin, rutin, and protocatechuic acid showed appreciable inhibitory effects.

## Figures and Tables

**Table 1:** The main activities of the investigated plants.

Plant	Popular Name	Biological Activity	References
*Melilotus officinalis* (MO)	Melilot, sweet clover	Used traditionally for the treatment of insect bite, circulatory disturbance in minor veins, liver disorders, hypertension, arthritis, hemorrhoids, and bronchitis	[4,5,6]
		Antimicrobial effects:	[7,8]
		Greater effect on Gram-positive bacteria than on the Gram-negative bacteria	[9]
*Coronilla varia* (CV)	Crown vetch	Cardiac, diuretic, purgative, diuretic heart tonic, antibacterial, and anticancer activities	[10,11,12]
		CV methanolic extract exhibited antibacterial activity against *S. pyogenes* (ATCC 19615), *S. aureus* (ATCC 25923), *S. epidermidis* (ATCC 12228), *P. aeruginosa* (ATCC 27853), *K. pneumoniae* (ATCC 13883), and *E. coli* (ATCC 25922) through the disc diffusion assay	[13]
*Ononis spinosa* (OS)	Spiny restharrow	Used for the urinary tract, kidney stones, inflammatory diseases, wound healing, skin disorders and/or infections, antibacterial, antifungal, anti-inflammatory, and analgesic effects.	[14,15]
		Aqueous extracts active against *S. Pyogenes* and Gram-positive microorganisms as *E. Coli, P. Aeruginosa, S. Typhimurium, S. Aureus,* and *C. Albicans*	[16,17]
*Robinia pseudoacacia* (RP)	Black locust	Antacid, antibacterial, antifungal, purgative effects and acts as an emmenagogue	[18]

**Table 2:** The individual profile of polyphenols detected using the LC method (mg·g^−1^).

Compound	Retention Time (min)	RP	MO	CV	OS
Gallic acid	4.8	0.693 ± 0.011 ^a^	nd	0.249 ± 0.006 ^b^	0.007 ± 0.0001 ^c^
Protocatechuic acid	10.8	0.701 ± 0.012 ^a^	0.696 ± 0.007 ^a^	0.155 ± 0.006 ^b^	0.011 ± 0.0003 ^c^
Caffeic acid	21.9	0.567 ± 0.008 ^a^	2.441 ± 0.03 ^b^	0.594 ± 0.008 ^a^	0.668 ± 0.013 ^c^
Epicatechin	22.7	17.002 ± 0.181 ^a^	65.879 ± 0.424 ^b^	2.219 ± 0.025 ^c^	nd
Coumaric acid	24.4	0.179 ± 0.004 ^a^	0.999 ± 0.014 ^b^	0.104 ± 0.002 ^c^	0.043 ± 0.001 ^d^
Ferulic Acid	24.7	nd	nd	nd	0.073 ± 2.76
Rutin	25.7	35.257 ± 2.84 ^a^	7.865 ± 0.71 ^b^	2.779 ± 44.42 ^c^	2.156 ± 60.88 ^d^
Rosmarinic acid	28.8	4.430 ± 0.43 ^a^	0.640 ± 21.12 ^b^	2.051 ± 38.98 ^c^	4.391 ± 115.79 ^a^
Resveratrol	31.9	2.176 ± 12.73 ^a^	1.518 ± 27.54 ^b^	1.256 ± 29.50 ^c^	1.107 ± 21.24 ^d^
Quercetin	32.1	1.786 ± 14.14 ^a^	nd	0.536 ± 5.50 ^b^	2.838 ± 50.54 ^c^
Kaempferol	34.9	0.669 ± 7.07 ^a^	1.114 ± 199.46 ^b^	1.878 ± 37.9 ^c^	4.861 ± 54.29 ^d^

The values are expressed as mean values ± standard deviations of all measurements. ^a–d^ *t*-test was used to compare the means differences registered among samples; data within the same row sharing different superscripts are significantly different (*p* < 0.05); data within the same row sharing the same superscripts are not significantly different (*p* > 0.05); nd—not detected.

**Table 3:** The efficiency of identified standards on the four sensitive strains selected.

Strain/Component	*E. coli*		*S. typhimurium*		*P. aeruginosa*		*S. pyogenes*	
	BGR (%)	BIR (%)	BGR (%)	BIR (%)	BGR (%)	BIR (%)	BGR (%)	BIR (%)
Gallic acid 500	63.02	36.97	54.04	45.95	51.28	48.71	81.83	18.16
Gallic acid 50	51.35	48.64	39.58	60.41	41.97	58.02	76.1	23.89
Protocatechuic acid 500	49.53	50.46	37.91	62.08	42.84	57.15	53.96	46.03
Protocatechuic acid 50	47.23	52.76	38.02	61.97	42.01	57.98	59.33	40.66
Caffeic acid500	54.89	45.1	47.36	52.63	44.52	55.47	49.81	50.18
Caffeic acid50	59.78	40.21	42.28	57.71	47.41	52.58	56.94	43.05
Epicatechin 500	58.89	41.1	43.2	56.79	45.27	54.72	61.58	38.41
Epicatechin 50	90.02	9.97	92.46	7.53	91.29	8.7	90.66	9.33
Coumaric acid 500	95.15	4.84	92.76	7.23	87.4	12.59	99.05	0.94
Coumaric acid 50	97.01	2.99	88.08	11.91	89.35	10.64	99.27	0.72
Ferulic acid 500	94.07	5.92	90.79	9.2	96.61	3.38	99.18	0.81
Ferulic acid 50	85.95	14.04	83.14	16.85	98.03	1.96	98.15	1.84
Rutin 500	77.1	22.89	52.3	47.69	52.25	47.74	66.18	33.81
Rutin 50	77.1	22.89	51.45	48.54	65.6	34.39	90.03	9.96
Rosmarinic acid 500	104.35	−4.35	86.02	13.97	91.71	8.28	94.95	5.04
Rosmarinic acid 50	123.05	−23.05	90.47	9.52	97.08	2.91	98.64	1.35
Resveratrol 500	113.57	−13.57	83.93	16.06	94.53	5.46	99.09	0.9
Resveratrol 50	66.83	33.16	51.06	48.93	77.99	22.01	90.3	9.69
Quercetin 500	87.12	12.87	44.27	55.72	67.44	32.55	63.88	36.11
Quercetin 50	93.53	6.46	52.56	47.43	88.43	11.56	90.53	9.46
Kaempferol 500	51.63	48.36	38.43	61.56	40.63	59.36	52.02	47.97
Kaempferol 50	94.17	5.82	85.06	14.93	93.01	6.98	84.62	15.37

**Table 4:** The MIC (µL/100 mL) for plant extracts CV, RP, MO, and OS.

	*S. pyogenes*	*S. aureus*	*P. aeruginosa*	*S. flexneri*	*E. coli*	*S. typhimurium*	*H. influenzae*	*C. parapsilopsis*	*C. albicans*
CV	25	25	25	25	25	25	25	25	25
CV	33	33	33	33	33	33	33	33	33
CV	40	40	40	40	40	40	40	40	40
RP	25	25	25	25	25	25	25	25	25
RP	33	33	33	33	33	33	33	33	33
RP	40	40	40	40	40	40	40	40	40
MO	25	25	25	25	25	25	25	25	25
MO	33	33	33	33	33	33	33	33	33
MO	40	40	40	40	40	40	40	40	40
OS	25	25	25	25	25	25	25	25	25
OS	33	33	33	33	33	33	33	33	33
OS	40	40	40	40	40	40	40	40	40

The samples that had no inhibition effect, causing a mass growth of the strain, are marked in dark grey color. The light gray color represents the samples in which the MIC was found, but subsequent concentrations showed a potentiating effect. Therefore, the effect decreased together with the concentration. The white color highlights the samples where the MIC was determined. The effect was maintained together with an increase in concentration.

**Table 5:** The MIC (µL/100 mL) for polyphenol standards against the most sensitive strains.

	*E. coli*	*S. typhimurium*	*P. aeruginosa*	*S. pyogenes*
Gallic acid	500	500	500	500
Gallic acid	50	50	50	50
Protocatechuic acid	500	500	500	500
Protocatechuic acid	50	50	50	50
Caffeic acid	500	500	500	500
Caffeic acid	50	50	50	50
Epicatechin	500	500	500	500
Epicatechin	50	50	50	50
Coumaric acid	500	500	500	500
Coumaric acid	50	50	50	50
Ferulic acid	500	500	500	500
Ferulic acid	50	50	50	50
Rutin	500	500	500	500
Rutin	50	50	50	50
Rosmarinic acid	500	500	500	500
Rosmarinic acid	50	50	50	50
Resveratrol	500	500	500	500
Resveratrol	50	50	50	50
Quercetin	500	500	500	500
Quercetin	50	50	50	50
Kaempferol	500	500	500	500
Kaempferol	50	50	50	50

The samples that had no inhibition effect, causing a mass growth, are marked in dark grey color. The light gray color represents the samples in which the MIC was found. However, subsequent concentrations showed a potentiating effect. Therefore, the effect decreased with the concentration. The white color highlights the samples in which the MIC was determined. The effect was maintained together with an increase in concentration.

**Table 6:** Correlations between variables (OD mean values of spectrophotometric determination of antimicrobial activity and the polyphenol concentration analyzed by LC).

	*S. pyogenes*	*S. aureus*	*P. aeruginosa*	*S. flexneri*	*E. coli*	*S. typhmurium*	*H. ifluezae*	*C. parpsilopsis*	*C. albcans*	Gallic acid	Protocatechuic acid	Caffeic acid	Epicatechin	Coumaric acid	Ferulic acid	Rutin	Rosma-rinic acid	Resvera-trol	Quercetin	Kaem-pferol
*S. pyogenes*	1																			
*S. aureus*	0.618	1.000																		
*P. aeruginosa*	0.614	0.984	1.000																	
*S. flexneri*	0.685	0.392	0.320	1.000																
*E. coli*	0.247	0.008	0.090	−0.029	1.000															
*S. typhimurium*	0.914	0.693	0.693	0.720	0.345	1.000														
*H. influenzae*	0.817	0.512	0.465	0.936	0.089	0.777	1.000													
*C. parapsilopsis*	0.715	0.444	0.366	0.988	−0.014	0.728	0.958	1.000												
*C. albicans*	0.795	0.517	0.429	0.920	−0.102	0.737	0.946	0.941	1.000											
Gallic acid	−0.025	−0.060	−0.037	−0.011	0.080	−0.045	0.064	−0.017	−0.023	1.000										
Protocatechuic acid	−0.258	−0.075	−0.018	−0.066	−0.014	−0.081	−0.082	−0.071	−0.185	0.437	1.000									
Caffeic acid	−0.280	−0.066	−0.035	−0.108	−0.137	−0.095	−0.211	−0.112	−0.212	−0.523	0.532	1.000								
Epicatechin	−0.302	−0.077	−0.033	−0.105	−0.108	−0.098	−0.190	−0.111	−0.226	−0.263	0.750	0.959	1.000							
Coumaric acid	−0.325	−0.100	−0.062	−0.137	−0.149	−0.129	−0.238	−0.144	−0.251	−0.375	0.659	0.986	0.990	1.000						
Ferulic acid	0.543	0.415	0.374	0.395	0.296	0.433	0.508	0.440	0.495	−0.472	−0.703	−0.290	−0.462	−0.428	1.000					
Rutin	0.044	0.081	0.119	0.107	0.188	0.095	0.206	0.118	0.067	0.896	0.699	−0.209	0.070	−0.065	−0.418	1.000				
Rosmarinc acid	0.526	0.359	0.341	0.380	0.371	0.393	0.568	0.420	0.475	0.484	−0.289	−0.794	−0.717	−0.790	0.542	0.434	1.000			
Resveratrol	−0.079	0.005	0.047	0.022	0.111	0.006	0.089	0.029	−0.035	0.848	0.826	−0.031	0.251	0.123	−0.574	0.977	0.233	1.000		
Quercetin	0.605	0.441	0.413	0.448	0.393	0.471	0.631	0.497	0.553	0.134	−0.472	−0.651	−0.667	−0.706	0.810	0.143	0.930	−0.062	1.000	
Kaempferol	−0.133	0.034	0.051	−0.006	−0.071	0.018	−0.090	−0.001	−0.081	−0.753	0.261	0.943	0.824	0.874	0.032	−0.435	−0.692	−0.298	−0.443	1.000

## Data Availability

The report of the analyzes performed for the samples in the paper can be found at the Interdisciplinary Research Platform (PCI) belonging to the Banat University of Agricultural Sciences and Veterinary Medicine "King Michael I of Romania" from Timisoara, being registered with number 39c/29.05.2019.

## Supplementary Materials

The following are available online at https://www.mdpi.com/article/10.3390/antibiotics10060662/s1, Table S1: The OD mean values of extracts at 540 nm.
