# Supplementary material for: Phytochemical Profile and Microbiological Activity of Some Plants Belonging to the Fabaceae Family
Source: Antibiotics (Basel). 2021 Jun 1;10(6):662. doi: 10.3390/antibiotics10060662 (PMC8227729; doi:10.3390/antibiotics10060662)
Supplement: Supplementary file 1 [file antibiotics-10-00662-s001.zip › antibiotics-1203166-supplementary.pdf]

Supplementary data

The OD mean values of extracts at 540 nm

	S. pyogenes	*S. aureus*	*P. aeruginosa*	*S. flexneri*	*E. coli*	*S.typhimurium*	*H. influenzae*	*C. parapsilopsis*	*C. albicans*
CV 25	0.277±0.003[a]	0.158±0.004[a]	0.149±0.003[a]	0.184±0.005[a]	0.199±0.004a	0.172±0.003[a]	0.199±0.004[a]	0.162±0.002[a]	0.215±0.007[a]
CV 33	0.254±0.003[b]	0.193±0.003[b]	0.198±0.003[b]	0.229±.003[b]	0.232±0.003[b]	0.184±0.002[b]	0.194±0,004[a]	0.195±0.005[b]	0.198±0.003[b]
CV 40	0.199±0,002[c]	0.275±0.004[c]	0.248±0.004[c]	0.271±0.005[c]	0.258±0.003[c]	0.229±0.004[c]	0.239±0,003[b]	0.249±0.002[c]	0.259±0.004[c]
RP 25	0.401±0.004[d]	0.245±0.004[d]	0.215±0.002[d]	0.242±0.002[d]	0.228±0.004[b]	0.235±0.007[c]	0.312±0,006[c]	0.244±0.002[c]	0.268±0.003[c,[illegible]]
RP 33	0.301±0,003[e]	0.272±0.003[c]	0.273±0.005[e]	0.285±0.003[e]	0.259±0.003[c]	0.265±0.003[d]	0.333±0,003[d]	0.296±0.003[d]	0.299±0.002[f]
RP 40	0.257±0,005[b]	0.403±0.005[e]	0.409±0.004[f]	0.418±0.003[f]	0.534±0.005[d]	0.443±0.008[e]	0.433±0,006[e]	0.456±0.004[e]	0.417±0.004[g]
MO 25	0.191±0,004[c]	0.167±0,003[f]	0.173±0.003[g]	0.161±0.004[g]	0.172±0,005[e]	0.164±0.001[f]	0.189±0,004[f]	0.183±0,003[f]	0.213±0.004[a]
MO 33	0.22±0.001[f]	0.254±0,004[d]	0.271±0.005[e]	0.283±0.006[c,e]	0.261±0,003[c]	0.255±0.004[g]	0.244±0,004[b]	0.242±0,003[c]	0.250±0.005[c,[illegible]]
MO 40	0.346±0,003[g]	0.392±0,003[g]	0.364±0.005[g]	0.379±0.004[h]	0.388±0,004[f]	0.374±0.007[h]	0.37±0,003[g]	0.413±0.005[g]	0.346±0.003[g]
OS 25	0.229±0,003[f]	0.22±0,004[h]	0.212±0.005[b,d]	0.231±0.002[b]	0.241±0,002[g]	0.222±0.005[c]	0.242±0,005[b]	0.228±0,002[h]	0.241±0.005[h]
OS 33	0.414±0,003[h]	0.335±0,003[i]	0.327±0.003[h]	0.343±0.005[i]	0.335±0,006[h]	0.319±0.003[i]	0.442±0,004[e]	0.379±0,003[i]	0.384±0.003[i]
OS 40	0.551±0,003[i]	0.533±0,003[j]	0.473±0.005[i]	0.504±0.006[j]	0.494±0,002[i]	0.58±0.004[j]	0.54±0,002[h]	0.576±0,003[j]	0.554±0.002[j]
**Control**	**0.448±0,001[j]**	**0.124±0,003[k]**	**0.282±0.002[j]**	**0.197±0.001[k]**	**0.592±0,002[j]**	**0.675±0.001[k]**	**0.698±0,001[i]**	**0.264±0,00[k]**	**0.243±0.005[h]**

The values are expressed as mean values ± standard deviations of all measurements. Different letter in the column indicates significant differences ($p < 0.05$) between values according to the t-test as different letters within the same column represent significant differences.
